# Dirac-Like Ferrimagnet Ce_3_Au_4_Ge_2_Bi_4_ as a Member of a Homologous Series of Square-Net Topological Materials

**DOI:** 10.1021/jacs.6c03149

**Published:** 2026-07-01

**Authors:** Atsushi Yamashita, Ryota Mizuno, Masayuki Ochi, Tatsuhiro Kojima, Hiraku Saito, Taro Nakajima, Akiko Nakao, Motoi Kimata, Masaki Kondo, Masashi Tokunaga, Takanori Kida, Masayuki Hagiwara, Masaki Nishi, Hiroshi Murakawa, Noriaki Hanasaki, Hideaki Sakai

**Affiliations:** † Department of Physics, The University of Osaka, Toyonaka, Osaka 560-0043, Japan; ‡ Forefront Research Center, The University of Osaka, Toyonaka, Osaka 560-0043, Japan; § Kobe City College of Technology, Kobe, Hyogo 651-2194, Japan; ∥ The Institute for Solid State Physics, The University of Tokyo, Kashiwa, Chiba 277-8581, Japan; ⊥ Comprehensive Research Organization for Science and Society (CROSS), Tokai, Ibaraki 319-1106, Japan; # Advanced Science Research Center, Japan Atomic Energy Agency, Tokai, Ibaraki 319-1195, Japan; ∇ Institute for Materials Research, Tohoku University, Sendai, Miyagi 980-8577, Japan; ○ Center for Advanced High Magnetic Field Science (AHMF), Graduate School of Science, The University of Osaka, Toyonaka, Osaka 560-0043, Japan; ◆ Spintronics Research Network Division, Institute for Open and Transdisciplinary Research Initiatives, The University of Osaka, Suita, Osaka 565-0871, Japan; □ RIKEN Center for Emergent Matter Science (CEMS), Saitama, 351-0198, Japan; ■ Institute of Materials Structure Science, High Energy Accelerator Research Organization, Ibaraki, 305-0801, Japan

## Abstract

Square-net compounds with the HfCuSi_2_-type framework have been a promising platform for realizing topological electronic states and their interplay with quantum many-body phenomena. In these materials, the transition-metal tetrahedral layer sandwiched between square-net layers plays a crucial role in determining the correlated physical properties, yet their structural variety has been limited to the single-layer type. Here, we report the discovery of Ce_3_Au_4_Ge_2_Bi_4_, a new square-net compound that represents the bilayer member of a homologous series bridging the HfCuSi_2_-type CeAuBi_2_ (112 phase) and ThCr_2_Si_2_-type CeAu_2_Ge_2_ (122 phase). The bilayer structure of Ce_3_Au_4_Ge_2_Bi_4_ is formed by combining two tetrahedral layers of the 112 phase through the insertion of a 122-like block, resulting in two mixed-anion antifluorite-type layers. Intriguingly, both the magnetic and electronic structures are substantially modified relative to the 112 phase. Single-crystal neutron diffraction reveals that the triple Ce layers coordinated by the tetrahedral bilayer exhibit a ferrimagnetic (up–down–up) order, which can be viewed as a combination of the antiferromagnetic orders in the 112 and 122 phases. First-principles calculations demonstrate that hybridization between Ce 5*d*-orbital in the spacer layer and Bi 6*p*-orbital in the square-net layer reshapes the band dispersion into a type-II Dirac-like band. Consistently, magnetotransport measurements show pronounced quantum oscillations with multiple frequencies and extremely light effective masses. These results establish Ce_3_Au_4_Ge_2_Bi_4_ as a prototype of a new homologous family of square-net-based topological magnets, where both magnetic order and Dirac-like band can be tuned by controlling the number of spacer layers. This tunable structural framework offers a strategy for the rational design of square-net materials with tailored functionalities.

## Graphical abstract

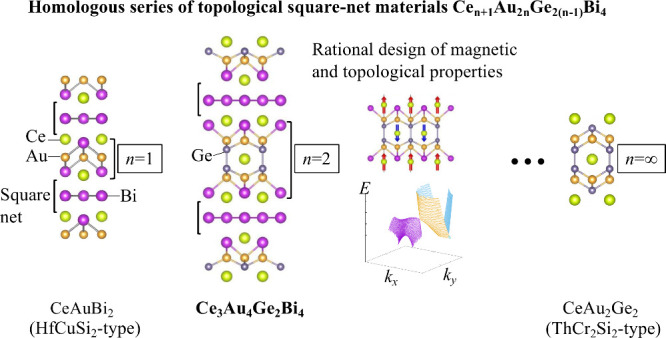

## Introduction

Materials with a square-net structure have recently attracted considerable attention as a promising platform for the exploration of topological quantum materials.^1–4^ The crystal structures of these materials are composed of alternating layers of square net and spacer layer; the former layer hosts topological electronic states (e.g., Dirac semimetal, nodal-line metal, and spin-Hall insulator), while the latter exhibits various many-body quantum phenomena (e.g., magnetic order, electric polarization, and superconductivity). This natural superlattice architecture enables the interplay of topological and strongly correlated phenomena, leading to potential application in spintronics and quantum computations.

The HfCuSi_2_-type structure is a prototypical example, incorporating square nets (or orthorhombically distorted variants) of group-IV/V elements (Figure 1b). The structure of spacer layer is the antifluorite-type, consisting of tetrahedral networks formed by transition metals and group-IV/V elements, coordinated by rare-earth ions. Strong electron correlations associated with transition metal and rare-earth in the spacer layer give rise to a wide range of emergent phenomena, including novel magnetic order^5–10^ and high-*T*_c_ superconductivity,^11,12^ which can coexist with topological electronic states originating from the square net. In several magnetic square-net compounds, recent experiments revealed distinct coupling between magnetic order and Dirac/Weyl-Fermion.^10,13–16^

**Figure 1:**
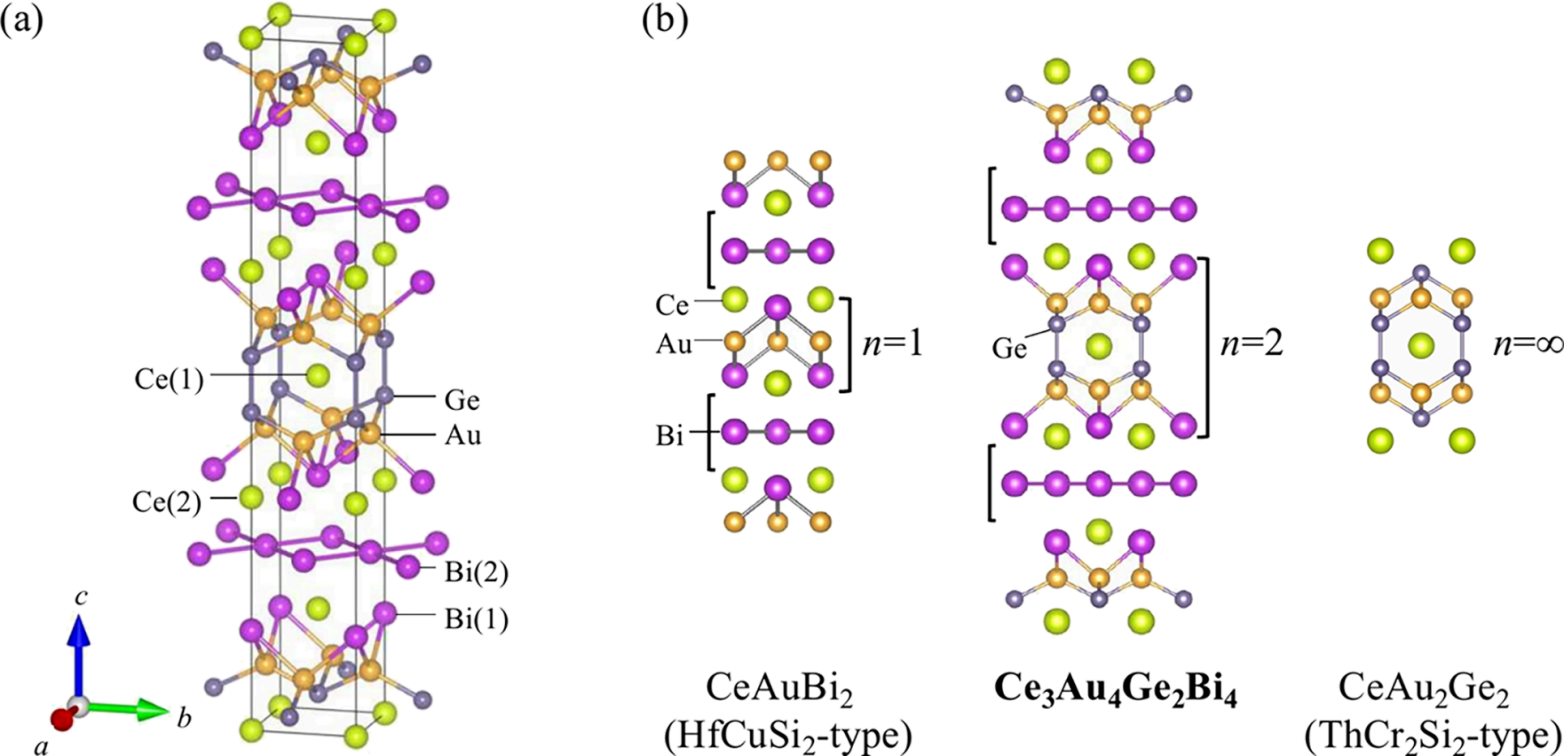
(a) Crystal structure of Ce_3_Au_4_Ge_2_Bi_4_. (b) Comparison of the layered structure of CeAuBi_2_ (left), Ce_3_Au_4_Ge_2_Bi_4_ (middle), and CeAu_2_Ge_2_ (right), representing the homologous series Ce_*n*+1_Au_2*n*_Ge_2(*n*–1)_Bi_4_, where *n* denotes the number of transition-metal tetrahedral layers sandwiched between Bi square nets.

Despite these rich physical properties, the structural diversity of the spacer layer for square-net compounds has remained largely confined to the HfCuSi_2_-type framework or its close derivatives.^17^ Expanding the range of spacer-layer structures while preserving the square-net framework is therefore an important challenge in materials development. Considering that the spacer layer of the HfCuSi_2_-type structure consists of a single tetrahedral layer (Figure 1b), systematical variation of the number of transition-metal tetrahedral layers could allow for constructing a homologous series of square-net materials. This concept is reminiscent of the Ruddlesden–Popper phases in perovskite-type oxides, where plenty of strongly correlated materials are rationally designed by tuning the number of transition-metal–oxygen layers.^18^ However, while a few related compounds with complex spacer layers have been reported,^17^ no derivatives of the HfCuSi_2_-type with variable tetrahedral-layer number (*n*) have been known to date. Here, we report the discovery of a new square-net material, Ce_3_Au_4_Ge_2_Bi_4_, in which the spacer layer is composed of a transition-metal tetrahedral bilayer (*n* = 2). This finding opens a route for the systematic exploration of square-net topological materials through controlled spacer-layer number.

## Results and Discussion

Figure 1a illustrates the structure of the new square-net material Ce_3_Au_4_Ge_2_Bi_4_, which crystallizes in a body-centered tetragonal lattice (space group *I*4/*mmm*, see Table 1 for the refined crystallographic data). As shown in Figure 1b, its structure features a bilayer spacer, in contrast to single-layer spacer of the HfCuSi_2_-type CeAuBi_2_.^7,19,20^ In CeAuBi_2_, a single Au–Bi tetrahedral layer coordinated by Ce atoms is sandwiched between Bi square-net layers, while in Ce_3_Au_4_Ge_2_Bi_4_, an additional Ce-coordinated tetrahedral layer is incorporated. Between these two tetrahedral layers, a Ge–Ce–Ge slab is inserted, resulting in a bilayer mixed-anion (Bi and Ge) antifluorite-type structure. In terms of the chemical composition, this layer extension corresponds to inserting a CeAu_2_Ge_2_ unit, allowing the general formula for the homologous series with *n* tetrahedral layers to be expressed as Ce_n+1_Au_2n_Ge_2(n–1)_Bi_4_. In the limit of *n* = *∞*, the Bi square nets disappear entirely, yielding CeAu_2_Ge_2_ with the known ThCr_2_Si_2_-type structure.^21–24^ This establishes a structural link between the so-called 112 (*n* = 1) and 122 (*n* = *∞*) phases in a single homologous framework. Ce_3_Au_4_Ge_2_Bi_4_, with *n* = 2, thus represents the first experimental realization of this series.

**Table 1:** Crystallographic Data for Ce_3_Au_4_Ge_2_Bi_4_ Near Room Temperature (Tetragonal *I*4/*mmm*)­^a^

atom	site	*g*	*x*	*y*	*z*	*U*_eq_ (Å^2^)
Bi(1)	4e	1	0	0	0.14421(5)	0.0110(5)
Bi(2)	4d	1	0	1/2	1/4	0.0088(5)
Ce(1)	2a	1	0	0	0	0.0098(7)
Ce(2)	4e	1	0	0	0.33665(6)	0.0089(5)
Au	8g	1	0	1/2	0.07858(4)	0.0178(5)
Ge	4e	1	0	0	0.45939(13)	0.0159(9)

a *a* = *b* = 4.5784(3), *c* = 29.993(3)­Å, *Z* = 2, *V* = 628.71(10)­Å^3^ *R*(*F*) = 0.048, *wR*(*F* ^2^) = 0.107, *S* = 1.19 [*I* > 2σ­(*I*)].

The increase in tetrahedral layers is anticipated to significantly influence the physical properties, since the spacer layer for Ce_3_Au_4_Ge_2_Bi_4_ is structurally equivalent to a unit cell of the ThCr_2_Si_2_ type, known as a fertile arena of strongly correlated phenomena.^25^ Indeed, as shown below, Ce_3_Au_4_Ge_2_Bi_4_ exhibits striking differences from CeAuBi_2_, not only in magnetic ordering of Ce sites, but also in Dirac-like electronic bands derived from the Bi square net.

Figure 2a presents the temperature (*T*) dependence of magnetization *M*(*T*) along the *c* axis at 0.1 T for Ce_3_Au_4_Ge_2_Bi_4_, in comparison with the related compounds CeAuBi_2_ and CeAu_2_Ge_2_. For CeAuBi_2_ and CeAu_2_Ge_2_, the antiferromagnetic order occurs at the Néel temperature *T*_N_, as indicated by a kink in the *M*(*T*) curves. In contrast, Ce_3_Au_4_Ge_2_Bi_4_ exhibits a ferromagnetic-like transition at the Curie temperature *T*_C_ ∼ 5.2 K, showing magnetic order of sensitive to the difference in spacer layer structure. For Ce_3_Au_4_Ge_2_Bi_4_, the temperature dependence of magnetic susceptibility χ­(*T*) at high temperatures well obeys the Curie–Weiss law (inset to Figure 2c), resulting in the positive Curie–Weiss temperature θ_CW_ = 27.2 K. The effective magnetic moment is estimated to be 2.58 μ_B_, close to that for the local Ce moment (2.54 μ_B_), indicating the Ce ions are almost localized. Figure 2b–d feature the detailed temperature dependence of magnetic and transport properties for Ce_3_Au_4_Ge_2_Bi_4_ near *T*_C_. Below *T*_C_, *M*(*T*) further increases at T*∼ 3.1 K with decreasing temperature, signifying a successive magnetic transition (Figure 2c). Correspondingly, the metallic in-plane resistivity ρ_*xx*_ exhibits clear kinks at *T*_C_ and T* (Figure 2b).

**Figure 2:**
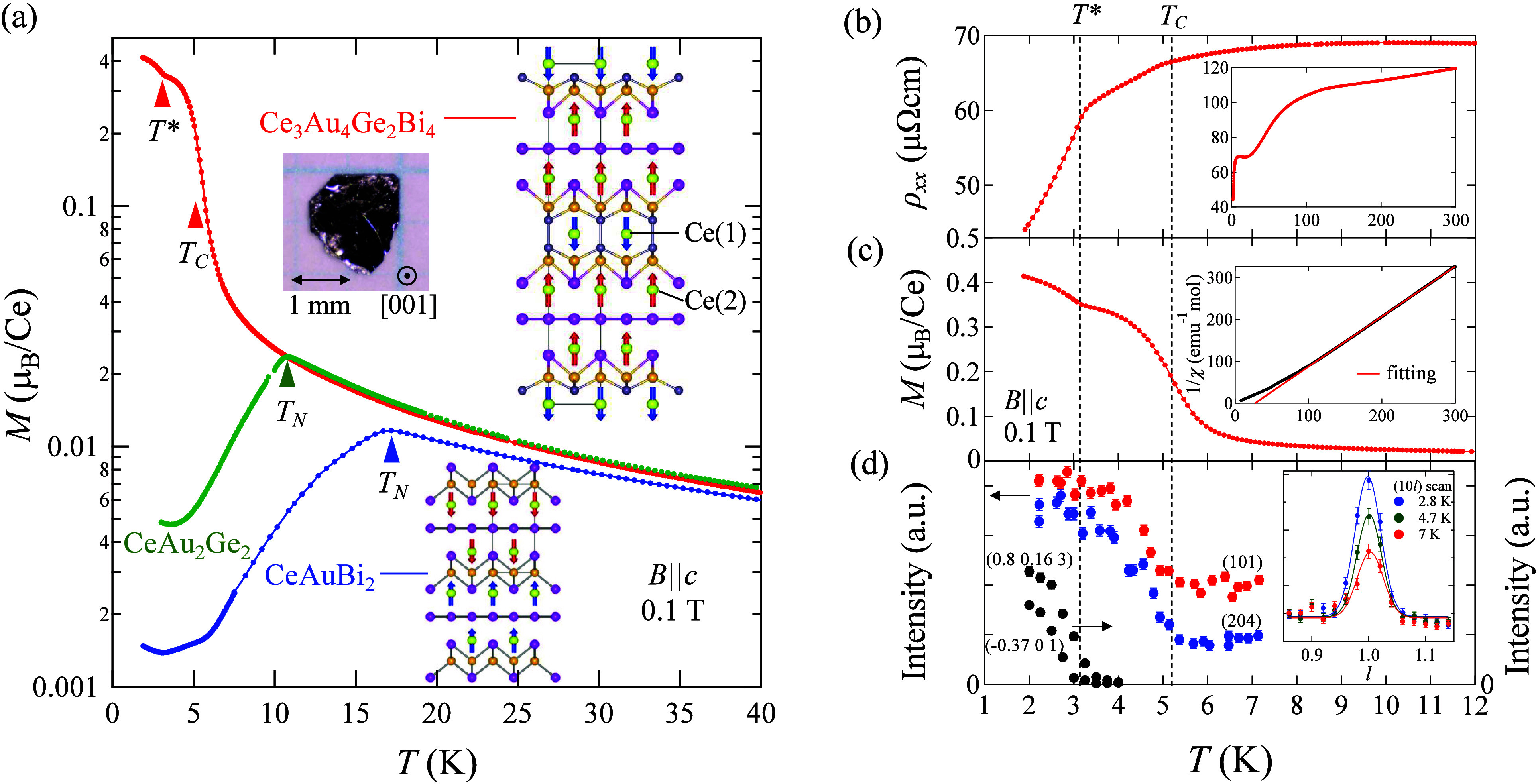
(a) Temperature (*T*) dependence of magnetization *M* measured at 0.1 T for *B* ∥ *c* for Ce_3_Au_4_Ge_2_Bi_4_ compared to related compounds CeAu_2_Ge_2_ and CeAuBi_2_. *T*_N_ (Néel temperature) denotes the antiferromagnetic transitions for CeAu_2_Ge_2_ and CeAuBi_2_, while *T*_C_ (Curie temperature) denotes the ferromagnetic-like transition for Ce_3_Au_4_Ge_2_Bi_4_. T* denotes the onset of subsequent transition. (b–d) *T* dependence of (b) resistivity ρ_*xx*_, (c) *M*, and (d) neutron diffraction intensities of selected reflections around *T*_C_ for Ce_3_Au_4_Ge_2_Bi_4_. In (d), the intensities of the commensurate magnetic reflections (101) and (204) (***q*** = 0) are shown, along with the incommensurate magnetic reflections (0.8 0.16 3) and (−0.37 0 1). Inset to (b) shows the overall *T* dependence of ρ_*xx*_ below 300 K. Inset to (c) shows the inverse susceptibility (1/χ­(*T*)), exhibiting the *T*-linear Curie–Weiss behavior at high temperatures. The red line denotes the fitted result obtained between 100 and 300 K. Inset to (d) shows the line scan along (1 0 *l*) at 2.8 K, 4.7 K (>T*), and 7 K (>*T*_C_).

The neutron diffraction measurements for a Ce_3_Au_4_Ge_2_Bi_4_ single crystal have revealed clear magnetic contributions superimposed on nuclear Bragg reflections, whose intensity evolves upon cooling, as seen in the (10*l*) line scan (inset to Figure 2d). These magnetic reflections appear only at nuclear Bragg positions, satisfying the reflection condition of *h* + *k* + *l* = 2*n* (*n* integer). The temperature dependence of intensities for the Bragg reflections (101) and (204) exhibit a sharp onset below *T*_C_ ∼ 5.2 K, consistent with the magnetization and resistivity (Figure 2d). At 2 K, we observed additional magnetic reflections with incommensurate modulation vectors (±0.63, 0, 0), (0, ±0.63, 0), (±0.20, ±0.16, 0), and (±0.16, ±0.2, 0) (Figure S1a–c). The intensity of these incommensurate magnetic reflections onsets below T* (Figure 2d), suggesting that the magnetic order at the ground state has multiple components that are spatially modulated within the *ab* plane. By contrast, the high-temperature phase (T* < *T* < *T*_C_) is characterized by a simple commensurate order with modulation vector (0, 0, 0). To determine the order of the Ce moments for the high-temperature phase, we have performed magnetic structural analyses based on diffraction data collected at 3.8 K (see Figure S1e for details). The best agreement between the observed and calculated structure factors was achieved by assuming the *c*-axis collinear ferrimagnetic structure model, in which the directions of the magnetic moments at Ce(1) and Ce(2) sites are opposite to each other. This results in an up–down–up stacking of two-dimensional ferromagnetic sheets as shown in the inset of Figure 2a. The amplitude of the ordered magnetic moment of Ce is estimated to be ∼1.0 μ_B_, leading to the net magnetization of $\frac{1}{3}\times 1.0\mu_{B}=0.33\mu_{B}$ per Ce for the ferrimagnetic state. This is consistent with the spontaneous magnetization observed at 4 K (∼0.32 μ_B_/Ce, inset to Figure 4a). Notably, due to the *c*-axis alignment, no magnetic intensity is predicted at the (00*l*) reflections, which is fully supported by the experimental data.

Here let us compare the ferrimagnetic order for Ce_3_Au_4_Ge_2_Bi_4_ with the antiferromagnetic orders for the related materials. In CeAuBi_2_, the Ce moments across the Bi square net are aligned ferromagnetically, whereas the Ce moments across the Au–Bi tetrahedral layer are aligned antiferromagnetically, resulting in an up–up-down–down order along the *c* axis^20^ (lower inset to Figure 2a). In Ce_3_Au_4_Ge_2_Bi_4_, the insertion of a CeAu_2_Ge_2_-like layer introduces a new Ce(1) site, inequivalent to the Ce(2) adjacent to the Bi square net (see Figure 1a). The observed up–down–up order indicates that Ce(1) and Ce(2) moments across the Au-(Ge/Bi) mixed-anion tetrahedral layer are coupled antiferromagnetically along the *c* axis, while Ce(2) moments across the Bi square net are coupled ferromagnetically (upper inset to Figure 2a). Such ferrimagnetic order can be understood as a natural combination of the magnetic orders present in CeAu_2_Ge_2_ (antiferromagnetic across the Au–Ge tetrahedral layer)^21,26^ and CeAuBi_2_ (ferromagnetic across the Bi square net).^20^

Nevertheless, *T*_C_ for Ce_3_Au_4_Ge_2_Bi_4_ is less than one-fifth of θ_CW_, implying the presence of magnetic frustration. Possible origins of this frustration include Ruderman-Kittel-Kasuya-Yosida (RKKY) interactions associated with Fermi surface nesting, as well as antiferromagnetic coupling between the second-nearest-neighbor Ce(2) sites across the tetrahedral bilayer. These interactions may compete with the nearest-neighbor antiferromagnetic coupling between Ce(1) and Ce(2) sites, giving rise to the complex incommensurate magnetic ground state, as observed experimentally. A detailed characterization of the incommensurate magnetic structure, however, is beyond the scope of the present study.

The new spacer layer in Ce_3_Au_4_Ge_2_Bi_4_ also strongly influences the electronic band structure. Figure 3a displays the overall band structure calculated based on the experimental lattice structure, where Ce-4*f* orbitals are treated as core states to simulate a nonmagnetic (paramagnetic) state (see Methods for details). As anticipated for the Bi square net structure [Bi(2) sites in Figure 1], highly dispersive linear bands are formed along the Γ-*X* and Σ-*Y* lines arising from the 6*p*_*x*_/6*p*_*y*_ orbitals. These bands cross the Fermi energy (*E*_F_ = 0), signifying significant contribution to the transport properties. What is distinct for the present material is that the crossing point of the linear bands along Γ-*X* is strongly deformed near *E*_F_ [denoted by a dotted circle in Figure 3a]. In Figure 3b,c, we show the detailed three-dimensional energy dispersion near *E*_F_ around this point on the *k*_*x*_–*k*_*y*_ plane. For the case without the spin–orbit coupling (SOC) [Figure 3b], the bands form so-called type-II Dirac point,^27–29^ where both upper and lower Dirac cones are significantly tilted to break the “Lorentz invariance”. When the SOC sets in [Figure 3c], although a gap opens at the Dirac point, the energy dispersion preserves the characteristic tilt as the hallmark of type-II Dirac cone.

**Figure 3:**
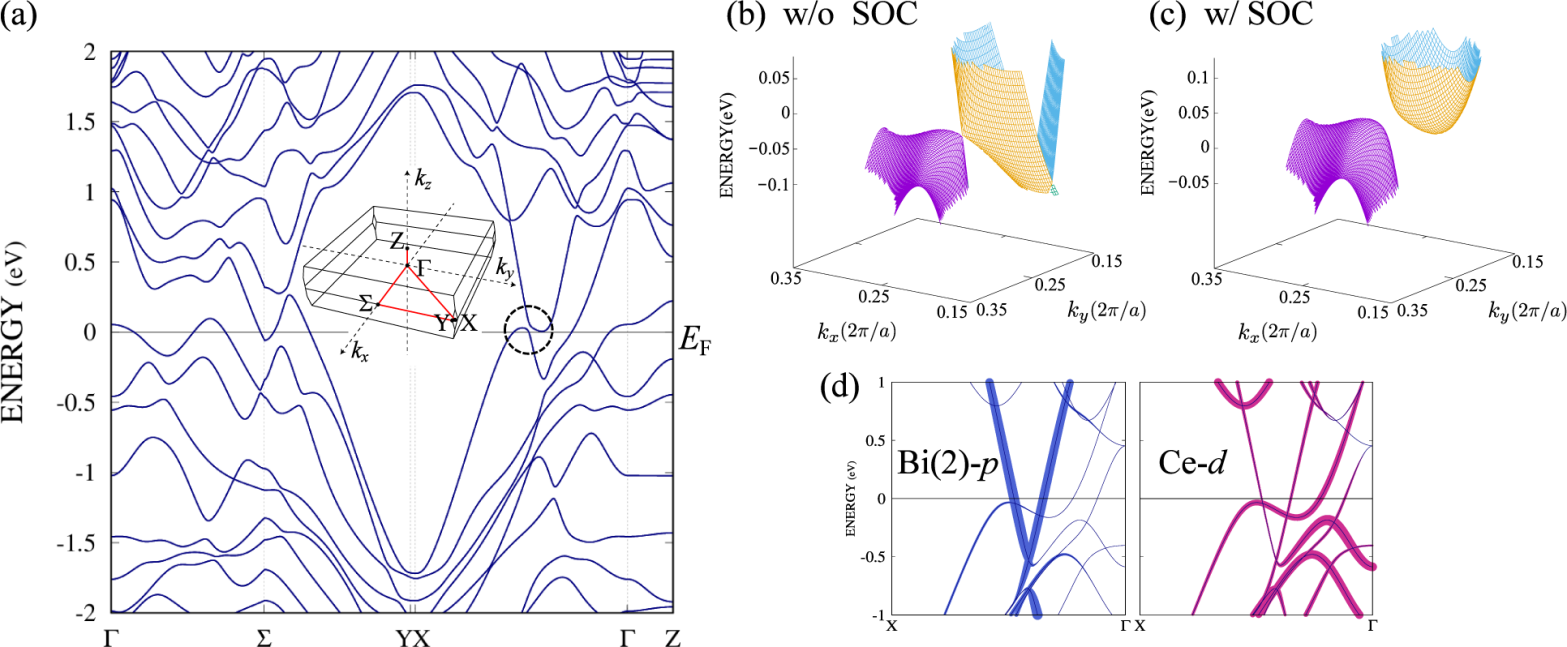
(a) Electronic band structure of Ce_3_Au_4_Ge_2_Bi_4_ calculated using the experimental crystal structure, including spin–orbit coupling (SOC). Ce-4*f* orbitals are treated as core states to simulate a nonmagnetic (paramagnetic) state. The dotted circle on the X−Γ line highlights the deformed Dirac point (gapped type-II Dirac point) formed near *E*_F_ (*E* = 0). The inset shows the Brillouin zone. (b, c) Three-dimensional energy dispersion on the *k*_*x*_–*k*_*y*_ plane around the type-II Dirac point [highlighted in panel (a)], calculated (b) without and (c) with SOC. (d) Electronic band dispersion with orbital character superimposed (without SOC). The thickness represents the weight of each orbital; Bi(2) *p* orbital (left) and Ce *d* orbital (right).

To investigate the underlying mechanism responsible for the strong deformation into type-II Dirac-like band, we have calculated the orbital-resolved band structure. Figure 3d presents the results without including SOC, which allows for a clearer identification of the orbital character of the bands without complex mixing by SOC. (The corresponding band structure with SOC is provided in Figure S2a,b) As shown in Figure 3d (left), Bi(2)-6*p* orbitals in the square-net layer form a well-defined Dirac band with large dispersion. Additionally, a less dispersive band derived from Ce-5*d* orbitals intersects above the Dirac point of the Bi(2)-derived bands, forming crossing points near *E*_F_ [Figure 3d (right)]. The crossing point closest to *E*_F_ corresponds to the type-II Dirac-like point, resulting from the significant difference in dispersion between the Bi 6*p* and Ce 5*d* orbitals of different parity. Importantly, the Ce(1) site in the inserted CeAu_2_Ge_2_-like layer significantly contributes to the Ce-5*d* band, indicating this additional spacer layer plays a key role in modifying the Dirac dispersion (see Figure S2c,d for details).

Finally, to experimentally verify the presence of such unconventional Dirac-like Fermions, we measured magnetotransport properties at low temperatures. Figure 4a,b show the field dependence of *M* and ρ_*xx*_ at 1.4 K (below T*) for ***B*** ||*c*, respectively. As the magnetic field increases, *M* exhibits a step-like jump at *B*_1_, followed by a transition into a forced ferromagnetic state at *B*_c_ (Figure 4a). Such multistep magnetic transitions are consistent with the complex magnetic ground state with multiple incommensurate modulation vectors. ρ_*xx*_ also strongly depends on the magnetic state, exhibiting a peak at *B*_1_, and then decreasing sharply above *B*_c_ (Figure 4b). In the high-field region (above ∼8 T), ρ_*xx*_ begins to gradually increase again and shows clear quantum oscillations, as highlighted in the inset of Figure 4b. The oscillatory component plotted as a function of 1/*B* is nonmonotonic with partially irregular spacing, indicating the presence of multiple frequency components (Figure 4c). Indeed, as shown in Figure 4d, the fast Fourier transform (FFT) analysis at 1.4 K reveals two dominant frequencies of *B*_F_ = 43 and 87 T, with a weaker peak at ∼160 T (Figure 4d). These FFT peak positions remain almost unchanged with increasing temperature, while the peak amplitudes gradually diminish and almost vanish near ∼30 K. The Lifshitz–Kosevich analysis of the main peak of 43 T (87 T) yields a small effective mass of m* = 0.12 *m*_0_ (0.27 *m*_0_) (Figure S3). The frequency of *B*_F_ = 43 T (87 T) corresponds to a Fermi surface cross-sectional area of *S*_F_ = 0.41 nm^–2^ (0.83 nm^–2^), which is less than 0.2% (0.5%) of the Brillouin zone area [(2π/4.53 Å)^2^ = 192 nm^–2^].

**Figure 4:**
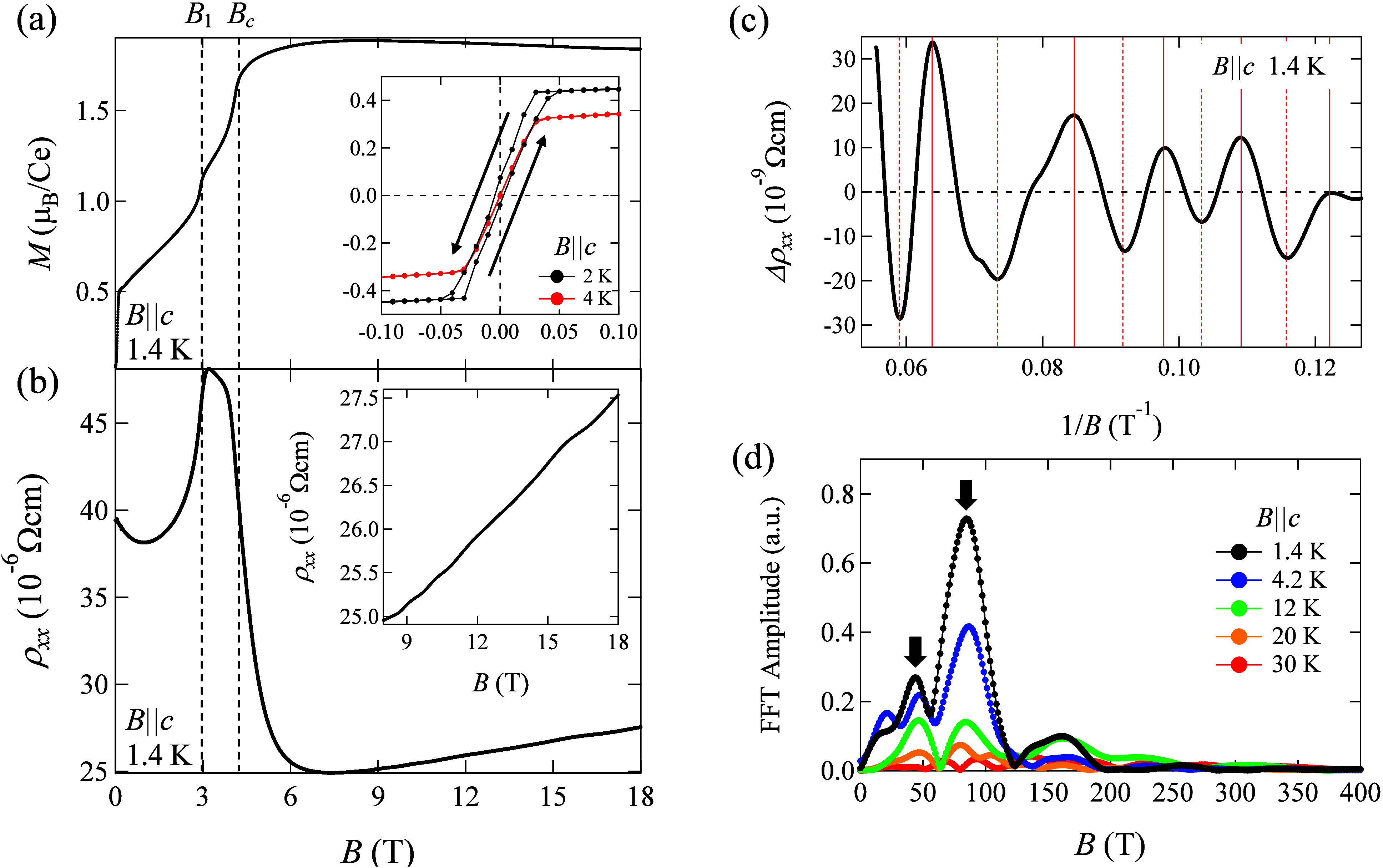
(a, b) Magnetic-field (*B*) dependence of (a) magnetization *M* and (b) resistivity ρ_*xx*_ at 1.4 K for *B* ∥ *c*. *B*_1_ and *B*_c_ denote the magnetic transition fields. Inset to (a) shows the *M*–*B* curves near zero field at 2 K (<T*) and 4 K (<*T*_C_), highlighting the spontaneous magnetization. Clear hysteresis was observed at 2 K. Inset to (b) shows the magnified profile at 8 *T* < *B* < 18 T, highlighting the oscillatory component of ρ_*xx*_. (c) Oscillatory component of ρ_*xx*_ (Δρ_*xx*_) as a function of 1/*B*, extracted from the ρ_*xx*_-*B* curve above *B*_c_ by subtracting a smooth monotonic background. (d) Fast Fourier transform (FFT) spectra of Δρ_*xx*_ at selected temperatures. Vertical arrows mark the dominant frequencies, which persist up to high temperatures.

For the assignment of the quantum oscillation frequencies, we compared them with the cross sections of the calculated Fermi surfaces. As a result, the two dominant frequencies (*B*_F_ = 43 and 87 T) can be uniquely assigned to the Fermi surface derived from the type-II Dirac-like band (the pocket highlighted in red in Figure S4a–c), as other conventional bands form much larger Fermi surfaces. On the other hand, the weaker peak at *B*_F_ = 160 T is likely associated with the smallest Fermi surface originating from the conventional bands (the pocket highlighted in green in Figure S4a–c). However, since several pockets with similar cross-sectional areas exist, this assignment is not unique at present.

We note that the quantum oscillations were measured in the forced ferromagnetic state, in which the type-II Dirac-like band remains present in the calculations, exhibiting spin splitting due to exchange interactions with the ferromagnetically aligned Ce moments (Figure S4b). The presence of multiple frequencies may arise from such spin-split Fermi surfaces and/or the *k*_*z*_ dispersion of the Fermi surface (Figure S4d). Alternatively, due to the strong tilting of the type-II Dirac cone, the electron-type pocket enclosing the Dirac point may cross *E*_F_ when the experimental *E*_F_ is shifted from *E* = 0. Thus, even considering the forced ferromagnetic state, the dominant frequencies (*B*_F_ = 43 and 87 T) can be reliably assigned to the type-II Dirac-like bands. However, the origin of the multiple frequencies remains tentative with several possible scenarios.

As for the topological properties of the electronic bands, a quantitative determination of the Berry phase from Landau fan analysis is not possible for the main quantum oscillations, owing to the multiple closely spaced frequency components and the weak two-dimensional character of the Fermi surface (Figure S5). Further experimental investigations, including angle-dependent quantum oscillation measurements^30^ and angle-resolved photoemission spectroscopy (ARPES), will be important for accurately determining the band structure and clarifying the detailed topological aspects. Taken together, the observed quantum oscillations indicate the presence of high-mobility carriers with a small Fermi surface and a light effective mass, consistent with the theoretically predicted Dirac-like bands. However, the detailed band dispersion and topological character cannot be fully resolved from the present experiments. Thus, Ce_3_Au_4_Ge_2_Bi_4_ provides a promising platform for exploring novel magnetotransport phenomena arising from the type-II Dirac-like band crossing *E*_F_.

## Conclusion

We propose a homologous series that bridges the HfCuSi_2_-type (*n* = 1) and ThCr_2_Si_2_-type (*n* = *∞*) structures, which enables the rational design of square-net-based topological materials through the variation in the spacer-layer structure. In this structural framework, square-net layers are separated by transition-metal tetrahedral layers coordinated by rare-earth ions, and systematic variation in the number of such spacer layers provides a structural and chemical design parameter for tuning the electronic and magnetic properties. Ce_3_Au_4_Ge_2_Bi_4_ (the “3424” phase) represents the first experimental realization of the bilayer member of this series, where both the magnetic order and Dirac-like band structure are strongly affected by the additional spacer layer. In particular, a strongly tilted type-II Dirac cone is predicted theoretically, while its nontrivial topological character remains to be established. Systematic variation in layer number thus expands not only the structural diversity but also the accessible range of physical properties and functionalities. Furthermore, the presence of four crystallographically distinct sites offers opportunities for chemical substitution, establishing a versatile platform for chemically designed square-net materials.

## Methods

### Crystal Growth

Single crystals of Ce_3_Au_4_Ge_2_Bi_4_ were grown by a Bi self-flux method. For Ce_3_Au_4_Ge_2_Bi_4_, high purity ingots of Ce (99.99%), Au (99.99%), Ge (99.999%), Bi (99.999%) were mixed in the ratio of Ce:Au:Ge:Bi = 3:6:2:60 and put into an alumina crucible in an argon-filled glovebox. The crucible was sealed in an evacuated quartz tube and heated at 1100 °C for 6 h, followed by slow cooling to 500 °C at the rate of ∼0.67 °C/h, where the excess Bi flux was decanted using a centrifuge. Plate-like single crystals with a typical size of ∼3 × 3 × 0.3 mm^3^ were obtained. Since these single crystals contained not only Ce_3_Au_4_Ge_2_Bi_4_ but also CeAuBi_2_, the target Ce_3_Au_4_Ge_2_Bi_4_ phase was identified by determining the *c*-axis length from the (00*l*) (*l* = 2*n*) reflections in their X-ray diffraction patterns. Powder X-ray diffraction measurements of the selected Ce_3_Au_4_Ge_2_Bi_4_ single crystals confirmed that they were single-phase without detectable impurities, except for a small amount of residual Bi flux on the surface.

The chemical composition of Ce_3_Au_4_Ge_2_Bi_4_ was determined by energy dispersive X-ray spectroscopy (EDX). The typical composition was found to be Ce_3_ *A*u_4.19(5)_Ge_2.31(4)_Bi_4.18(5)_, which agrees well with the stoichiometric ratio.

### Single-Crystal X-ray Diffraction and Crystal Structure Analysis

The diffraction data were collected near room temperature using a RIGAKU XtaLAB Synergy Custom diffractometer equipped with Hypix-6000HE detector with Mo*K*α radiation. A block shaped black shiny single crystal with the dimensions of ∼0.5 × 0.3 × 0.03 mm^3^ was mounted on a glass capillary.

The diffraction data of 1116 were collected and merged to give 284 unique reflections. The crystal structure (Table 1) was solved by a direct method and refined on *F* ^2^ by a least-squares method by the programs SHELXS^31^ and SHELXL-2018/3,^32^ respectively. All atoms were refined anisotropically. The final *R* values on 272 unique reflections with *I* > 2σ­(*I*) are 0.048 and 0.107 for *R*(*F*) and *wR*(*F* ^2^), respectively.

### Neutron Diffraction and Magnetic Structure Analysis

Neutron diffraction experiments were carried out on the polarized neutron triple-axis spectrometer (PONTA) at 5G beamline of Japan Research Reactor 3 (JRR-3) in Japan. The spectrometer was operated in the unpolarized two-axis mode with the horizontal beam collimation of open—80′–80′. An unpolarized monochromatic neutron beam with the energy of 34.05 meV was obtained using a pyrolytic graphite (PG) monochromator. The single crystal sample (with a dimension of ∼3 × 3 × 0.2 mm^3^) was loaded into a ^4^He cryostat with the (*h*, 0, *l*) scattering plane.

For the magnetic structure analysis, we measured integrated intensities of Bragg reflections (*h* 0 *l*), where *h* and *l* were integers. The magnetic intensities were extracted by subtracting the nuclear contribution measured at 10 K (>*T*_C_). Assuming the ferrimagnetic order shown in Figure 2a, we obtained good agreement between the observed and calculated magnetic structure factors with reasonable reliability factors of *R* = 10.0% and ordered moments *m*_Ce_ = 1.01 μ_B_ at 3.8 K. For more details of the analysis, see Figure S1.

We also carried out neutron experiments using the time-of-flight diffractometer SENJU (BL18) at the Materials and Life Science Experimental Facility (MLF) of the Japan Proton Accelerator Research Complex (J-PARC). The wavelength range of incident neutrons was selected to be 0.4–8.8 Å.

### Magnetic and Transport Measurements

The temperature (2–300 K) and field (below 7 T) dependences of magnetization were measured using Magnetic Property Measurement System (MPMS, Quantum Design) with Reciprocating Sample Option (RSO). The field dependence of magnetization up to 18 T was measured by the induction method, using coaxial pickup coils using the nondestructive pulsed magnet with a pulse duration of 36 ms at the International Mega-Gauss Science Laboratory at the Institute for Solid State Physics.

In-plane resistivity ρ_*xx*_ was measured by a conventional 4-terminal method with electrodes formed by room-temperature curing silver paste. The typical sample size was 1 × 0.3 × 0.05 mm^3^. The temperature dependence measured using Physical Properties Measurement Systems (PPMS, Quantum Design). The measurement up to 18 T was performed using a 18T superconducting magnet at High Field Laboratory for Superconducting Materials in Institute for Materials Research, Tohoku University. For resistivity measurements, we used a 4-probe AC method with a typical current and frequency of 1–5 mA and 20–150 Hz, respectively.

### First-Principles Calculations

First-principles calculations based on density functional theory (DFT) were performed using the projector-augmented wave (PAW) method^33^ as implemented in Vienna ab initio simulation package.^34–37^ We used the generalized gradient approximation with the Perdew–Burke–Ernzerhof (PBE) parametrization.^38^ In the ferromagnetic calculation, Ce, Au, Ge, and Bi atoms were described using PAW potentials with [Kr 4*d* ^10^]-core, [Xe 4*f* ^14^]-core, [Ar 3*d* ^10^]-core, and [Xe 4*f* ^14^5*d* ^10^]-core, respectively. In the nonmagnetic calculation, we used an open-core Ce potential, in which the 4*f* ^1^ electron is included in the core ([Kr 4*d* ^10^4*f* ^1^]-core). Plane-wave cutoff energy of 350 eV and a 12 × 12 × 12 *k* mesh were used. The experimental crystal structure shown in Table 1 was used for our DFT calculations. We also applied +*U* correction^39^ with the simplified rotationally invariant approach^40^ to the Ce-*f* orbitals with *U* = 5 eV in the ferromagnetic calculation. We constructed the tight-binding model for obtaining the Fermi surface by using the Wannier90 ^41^ software. As the basis functions of the tight-binding model, we extracted the maximally localized Wannier functions of the *s* and *d* orbitals of Au, *p* orbitals of Bi and Ge atoms. In addition, we included Ce-*d* and *f* orbitals (*d* orbitals) in the Wannier functions for the ferromagnetic (nonmagnetic) calculation. Fermi surfaces were depicted using the FermiSurfer^42^ software.

## Supplementary Material



## Notes

Details of neutron diffraction and magnetic structure analysis, band calculation including SOC, temperature dependence of quantum oscillation, calculated Fermi surface, and Landau fan diagram (PDF)
