# Supplementary material for: Dirac-Like Ferrimagnet Ce3Au4Ge2Bi4 as a Member of a Homologous Series of Square-Net Topological Materials
Source: J Am Chem Soc. 2026 Jul 1;148(27):28302–9. doi: 10.1021/jacs.6c03149 (PMC13383746; doi:10.1021/jacs.6c03149)
Supplement: Supplementary file 1 [file ja6c03149_si_001.pdf]

Supporting Information : "A Dirac-like ferrimagnet $Ce_3Au_4Ge_2Bi_4$ as a member of a homologous series of square-net topological materials"

Atsushi Yamashita,[1] Ryota Mizuno,[2] Masayuki Ochi,[1,2] Tatsuhiro Kojima,[3] Hiraku Saito,[4] Taro Nakajima,[4,5,6] Akiko Nakao,[7] Motoi Kimata,[8,9] Masaki Kondo,[4] Masashi Tokunaga,[4] Takanori Kida,[10] Masayuki Hagiwara,[10] Masaki Nishi,[1] Hiroshi Murakawa,[1] Noriaki Hanasaki,[1,11] and Hideaki Sakai[9,1,*]

[1]*Department of Physics, The University of Osaka, Toyonaka, Osaka 560-0043, Osaka*

[2]*Forefront Research Center, The University of Osaka, Toyonaka, Osaka 560-0043, Japan*

[3]*Kobe City College of Technology, Kobe, Hyogo 651-2194, Japan*

[4]*The Institute for Solid State Physics, The University of Tokyo, Kashiwa, Chiba 277-8581, Japan*

[5]*RIKEN Center for Emergent Matter Science (CEMS), Saitama 351-0198, Japan*

[6]*Institute of Materials Structure Science, High Energy Accelerator Research Organization, Ibaraki 305-0801, Japan*

[7]*Comprehensive Research Organization for Science and Society (CROSS), Tokai, Ibaraki 319-1106, Japan*

[8]*Advanced Science Research Center, Japan Atomic Energy Agency, Tokai, Ibaraki 319-1195, Japan*

[9]*Institute for Materials Research, Tohoku University, Sendai, Miyagi 980-8577, Japan*

[10]*Center for Advanced High Magnetic Field Science (AHMF), Graduate School of Science, The University of Osaka, Toyonaka, Osaka 560-0043, Japan*

[11]*Spintronics Research Network Division, Institute for Open and Transdisciplinary Research Initiatives, The University of Osaka, Suita, Osaka 565-0871, Japan*

I. DETAILS OF NEUTRON DIFFRACTION AND MAGNETIC STRUCTURE ANALYSIS

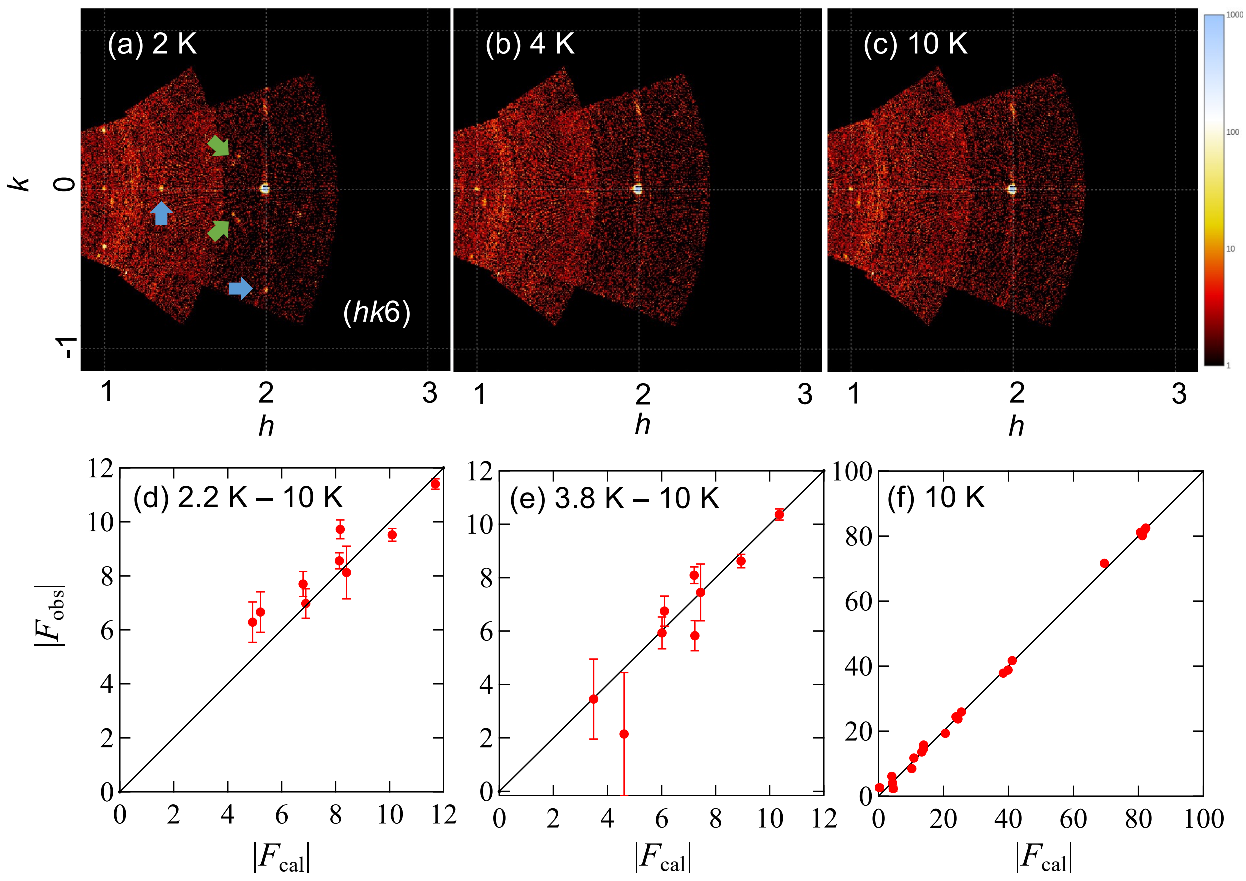

FIG. S1: (a-c) Neutron scattering intensity maps on the $(h\,k\,6)$ plane at (a) 2 K, (b) 4 K, and (c) 10 K. Incommensurate magnetic reflections are denoted by arrows in (a). (d, e) Comparison between calculated ($F_{\rm cal}$) and observed ($F_{\rm obs}$) magnetic structure factors at (d) 2.2 K and (e) 3.8 K, assuming the ferrimagnetic order shown in Fig. 2a of the main text. Magnetic intensities were obtained by subtracting the nuclear contribution measured at 10 K (above $T_{\rm C}$). (f) Comparison of calculated and observed nuclear structure factors at 10 K.

Figures S1(a)-(c) present neutron scattering intensity maps on the $(h\,k\,6)$ plane centered at $(2\,0\,6)$, measured at 0 T and temperatures of 2 K (below T^*), 4 K (above T^* below $T_{\rm C}$), and 10 K (above $T_{\rm C}$), respectively. Below $T_{\rm C}$ (but above T^*), magnetic reflections are superimposed on the nuclear Bragg reflections. Upon cooling below T^*, additional incommensurate magnetic reflections manifest themselves, with modulation vectors $(\pm 0.63, 0, 0)$,

$(0, \pm 0.63, 0)$, $(\pm 0.20, \pm 0.16, 0)$, and $(\pm 0.16, \pm 0.20, 0)$ [indicated by arrows in Fig. S1(c)]. Figures S1(d) and S1(e) compare the observed and calculated magnetic structure factors at 2.2 K (below T^*) and 3.8 K (above T^* below T_C), respectively, assuming the ferrimagnetic order shown in Fig. 2a of the main text. Good agreement is obtained with reasonable reliability factors of $R = 10.0\%\,(9.2\%)$ and ordered moments $m_{Ce} = 1.01\mu_B\,(1.14\mu_B)$ at 3.8 K (2.2 K). At 2.2 K, despite the emergence of incommensurate reflections, the result remains consistent with a ferrimagnetic structure, suggesting that the fundamental magnetic order is ferrimagnetic, which is partially modulated with long-period incommensurate components. Figure S1(f) shows the comparison of nuclear structure factors at 10 K (above T_C), based on the crystal structure determined by single-crystal X-ray diffraction (Table 1 in the main text). The result also shows excellent agreement, with a reliability factor of $R = 2.83\%$, further confirming the accuracy of the newly determined structure of $Ce_3Au_4Ge_2Bi_4$.

II. BAND CALCULATION INCLUDING SOC

Figures S2(a) and S2(b) show the orbital-resolved band structure including SOC, for (a) Bi(2)-p orbitals and (b) the Ce-d orbitals. Although SOC induces partial mixing of the orbital characters, the dominant contributions from each orbital remain essentially the same as in the calculations without SOC.

Figures S2(c) and S2(d) show the orbital weight originating from the crystallographic Ce(1) and Ce(2) sites, respectively. Notably, the Ce(1) site, characteristic of the new spacer structure, has a considerable contribution to the bands forming the type-II Dirac points.

III. TEMPERATURE DEPENDENCE OF QUANTUM OSCILLATION

We estimated the effective masses (m^*) corresponding to the main quantum-oscillation frequencies (B_F = 43 T and 87 T) from the temperature dependence of the oscillation amplitude (Fig. S3b). Conventional Lifshitz–Kosevich analysis yields $m^* = 0.12\,m_0$ for $B_F = 43$ T and $m^* = 0.27\,m_0$ for $B_F = 87$ T.

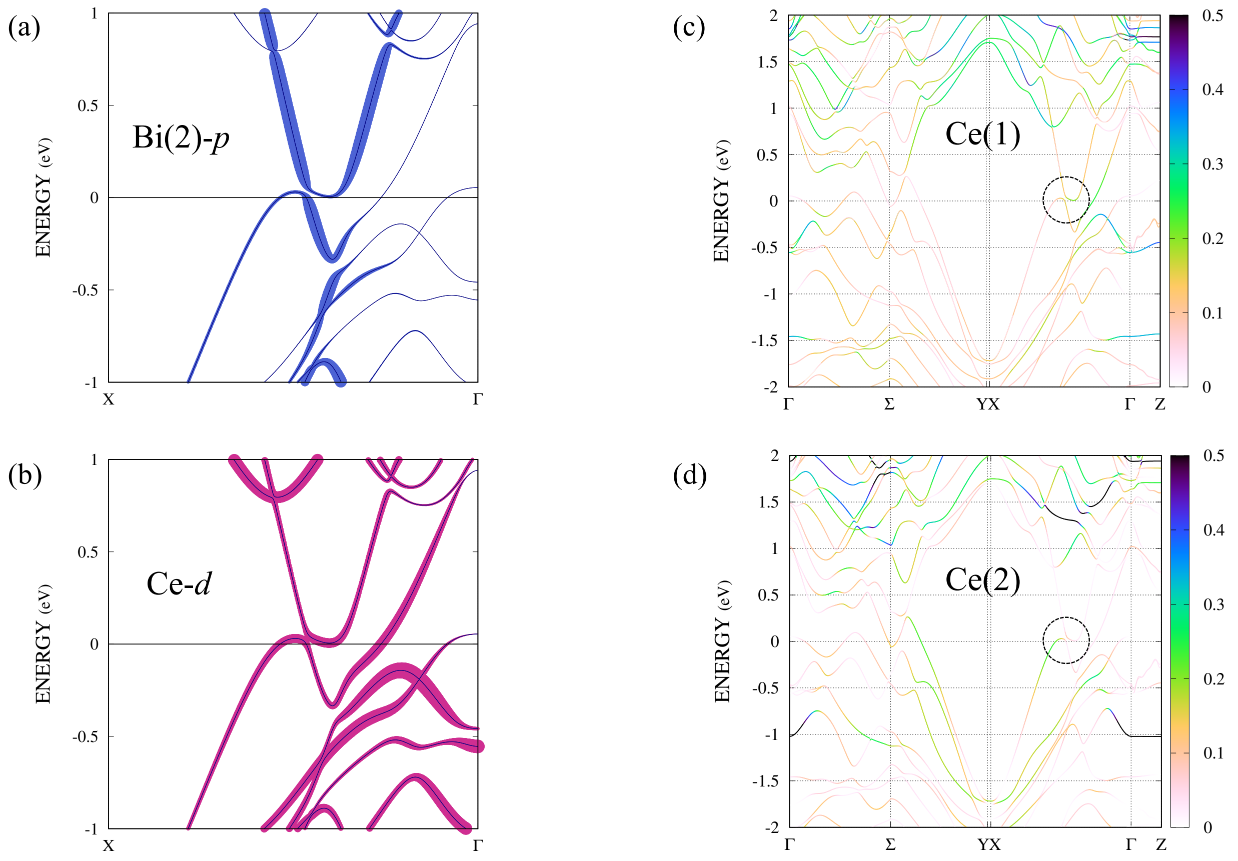

FIG. S2: (a, b) Band dispersion calculated with SOC. The thickness of the curves represents the orbital weight for (a) Bi(2)-p orbitals and (b) Ce-d orbitals. (c, d) Band dispersion calculated with SOC. The color scale represents the Ce orbital weight from each crystallographic site: (c) Ce(1) and (d) Ce(2).

IV. CALCULATED FERMI SURFACE

As shown in Fig. S4, the calculated Fermi surface for the present material consists of a couple of large sheet derived primarily from the Ce-Au atoms in the spacer layer. Among them, a small pocket is formed along the Γ-X line [denoted by the red circle in Figs. S4(a) and (c)], which corresponds to the quasi-2D cylindrical Fermi surface derived from Bi square net [Bi(2)] that hosts the type-II Dirac point. The calculated cross-sectional area S_F of this pocket as a function of k_z agrees well with the experimentally determined main frequencies of the quantum oscillation (B_F=43, 87 T) [Fig. S4(d)]. Another candidate of small pocket is a 3D spherical Fermi surface located along the Γ-Σ line [denoted by the green circle in Figs.

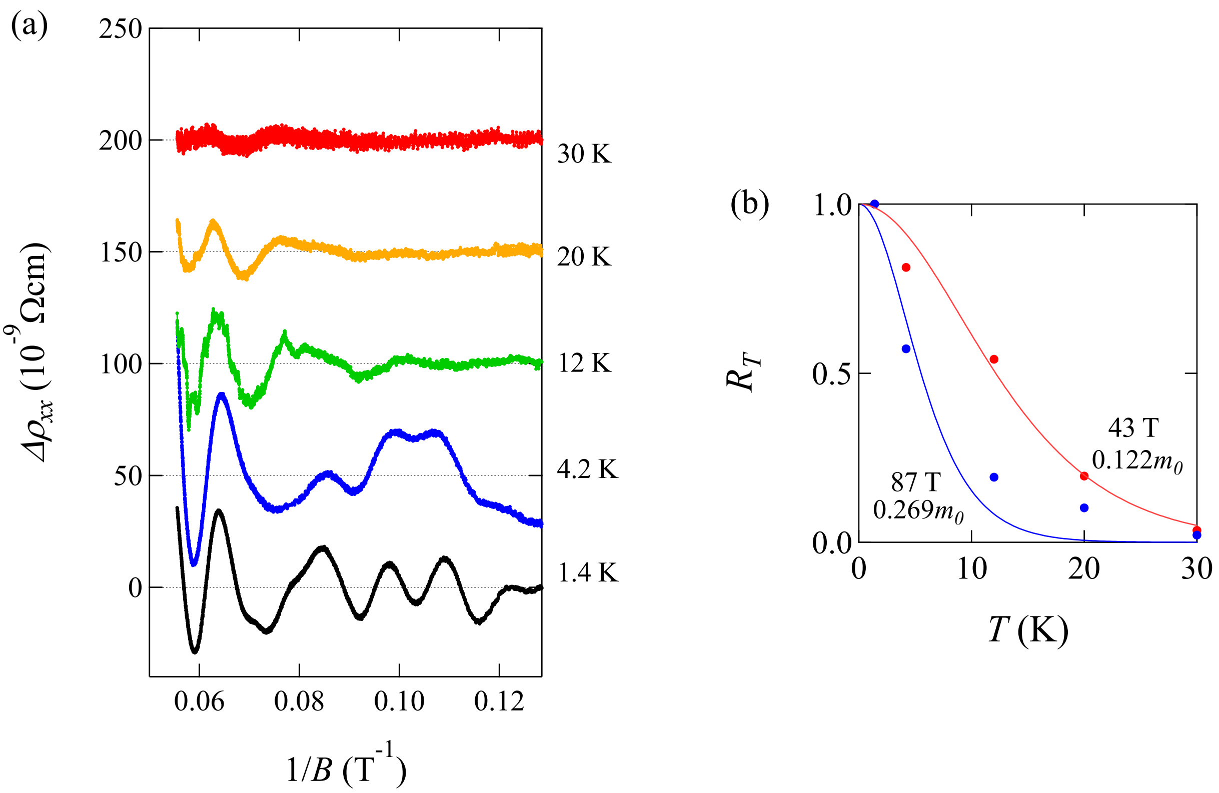

FIG. S3: (a) Oscillatory component of ρ_{xx} as a function of $1/B$ at various temperatures. Each curve is shifted vertically for clarity. (b) Magnitude of the FFT for each frequency (B_{F} = 43 T and 87 T) as a function of temperature. The solid curves represent fits based on the Lifshitz–Kosevich analysis.

S4(a) and (c)], mainly derived from Ce-Au atoms. The calculated S_F of this 3D pocket is larger than that of the cylindrical Fermi surface and roughly matches B_F = 160 T, which appears as a weak peak in the FFT of the quantum oscillation.

It is important to note that the quantum oscillations were observed in the forced ferromagnetic phase above B_c, where the spin-splitting of the bands are caused by the exchange coupling with the Ce-4f moments. To estimate the magnitude of spin-splitting, we also calculated the band structure in the forced-ferromagnetic phase. As shown in Fig. S4(b), except for weak splitting, the overall feature of the Fermi surface remains similar to that of the nonmagnetic calculation (i.e., with open-core Ce-f orbitals). Thus, regardless of the spin-splitting, the main quantum oscillation (B_F=43, 87 T) can be attributed to the cylindrical Fermi surface associated with the type-II Dirac point.

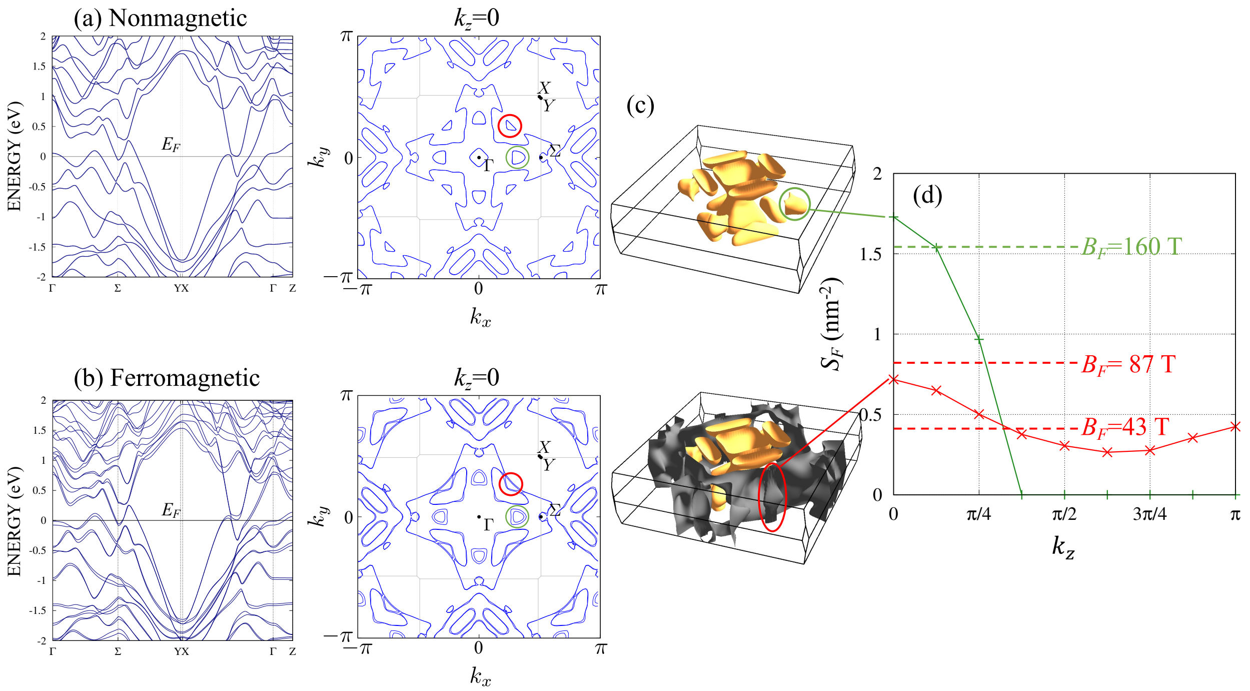

FIG. S4: (a, b) Calculated band structures (left) and Fermi-surface cross sections at $k_z = 0$ (right) for (a) the nonmagnetic state with open-core Ce-f orbitals and (b) the ferromagnetic state with fully aligned Ce-f moments. (c) Calculated Fermi surfaces for the nonmagnetic state. In the upper panel, the large black-colored Fermi sheets are omitted to show the inner sheet of the Fermi surface. (d) k_z dependence of the cross-sectional areas S_F for the selected Fermi surfaces indicated in panel (c). The red points correspond to the hole-type quasi-2D cylindrical Fermi surface associated with the type-II Dirac point, while the green points correspond to the small hole-type three-dimensional spherical pocket along the Γ-Σ line. Horizontal dashed lines indicate the experimental B_F values extracted from the FFT spectra of the quantum oscillations.

V. LANDAU FAN DIAGRAM

To perform the Landau fan analysis, we calculate the conductivity $\sigma_{xx} = \frac{\rho_{xx}}{\rho_{xx}^2+\rho_{yx}^2}$ and extract the oscillatory component $\Delta\sigma_{xx}$. Similar to $\Delta\rho_{xx}$, the $\Delta\sigma_{xx}$ plotted as a function of $1/B$ is non-monotonic with partially irregular spacing owing to the presence of multiple closely spaced frequencies (Fig. S5a). Nevertheless, in the field range where the $B_F = 87$ T component dominates, we analyze the data following the quantum Hall convention (i.e., assigning the peak (dip) positions to integer (half-integer) Landau indices N), yielding an

intercept close to zero and suggesting a nontrivial Berry phase $\phi_{\mathrm{B}} \sim \pi$ (Fig. S5b).

On the other hand, performing the same analysis using $\Delta\rho_{xx}$ results in an intercept close to 0.5, corresponding to $\phi_{\mathrm{B}} \sim 0$. These two approaches typically yield consistent results in related materials (Refs. [14] and [30] in the main text), as well as in conventional quantum Hall systems. In the present system, however, the coexistence of complex three-dimensional Fermi surfaces likely affects the scattering processes, making it difficult to unambiguously determine the Berry phase from the resistivity data alone.

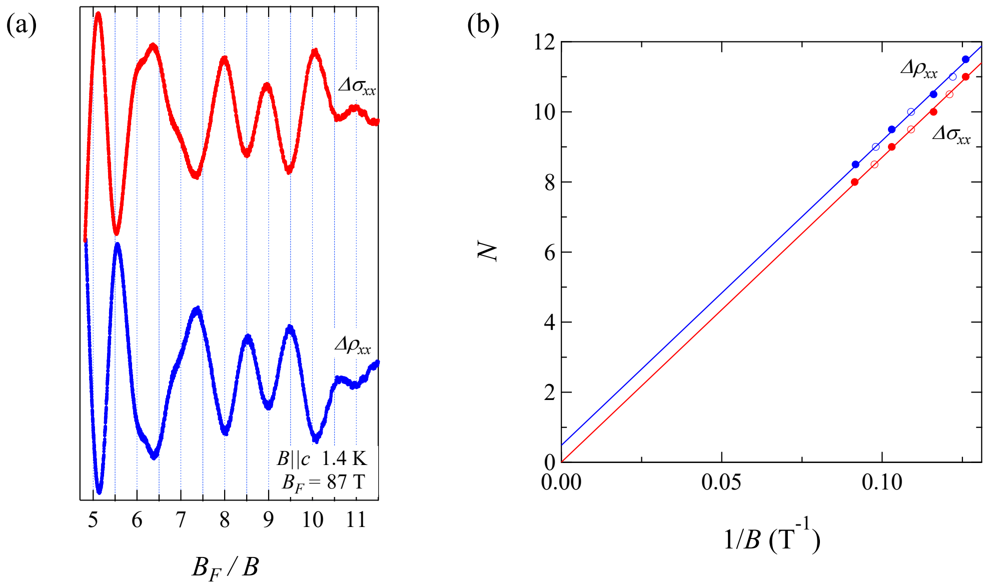

FIG. S5: (a) Oscillatory components of the resistivity ($\Delta\rho_{xx}$) and conductivity ($\Delta\sigma_{xx}$) at 1.4 K as a function of B_F/B ($B_F = 87$ T). (b) Landau fan plot (Landau index N versus $1/B$) obtained from $\Delta\rho_{xx}$ and $\Delta\sigma_{xx}$. The peak (dip) positions of the oscillations correspond to integer (half-integer) Landau indices. In this convention, the intercept on the N axis gives $\frac{1}{2} - \frac{\phi_{\mathrm{B}}}{2\pi}$, where ϕ_{B} is the Berry's phase.

---

* Corresponding author: `hideaki.sakai@tohoku.ac.jp`
